# Self-Supported Ni(P, O)_x_·MoO_x_ Nanowire Array on Nickel Foam as an Efficient and Durable Electrocatalyst for Alkaline Hydrogen Evolution

**DOI:** 10.3390/nano7120433

**Published:** 2017-12-06

**Authors:** Wei Hua, Huanyan Liu, Jian-Gan Wang, Bingqing Wei

**Affiliations:** 1 State Key Laboratory of Solidification Processing, Center for Nano Energy Materials, School of Materials Science and Engineering, Northwestern Polytechnical University and Shaanxi Joint Lab of Graphene (NPU), Xi’an 710072, China; huawei@mail.nwpu.edu.cn (W.H.); liuhuanyan@mail.nwpu.edu.cn (H.L.); 2 Department of Mechanical Engineering, University of Delaware, Newark, DE 19716, USA

**Keywords:** Ni(P, O)_x_·MoO_x_ nanowire array, synergistic effect, electrocatalyst, alkaline hydrogen evolution reaction

## Abstract

Earth-abundant and low-cost catalysts with excellent electrocatalytic hydrogen evolution reaction (HER) activity in alkaline solution play an important role in the sustainable production of hydrogen energy. In this work, a catalyst of Ni(P, O)_x_·MoO_x_ nanowire array on nickel foam has been prepared via a facile route for efficient alkaline HER. Benefiting from the collaborative advantages of Ni(P, O)_x_ and amorphous MoO_x_, as well as three-dimensional porous conductive nickel scaffold, the hybrid electrocatalyst shows high catalytic activity in 1 M KOH aqueous solution, including a small overpotential of 59 mV at 10 mA cm^−2^, a low Tafel slope of 54 mV dec^-1^, and excellent cycling stability.

## 1. Introduction

Exploring new sustainable energy resources and clean energy carriers to replace the traditional fossil fuels is one of the most important challenges of the 21st century. Hydrogen is considered as the most promising energy carrier for sustainable energy applications due to its outstanding energy storage density, environmental friendliness, and renewability [1,2,3]. Electrochemical water splitting is an important component of several hydrogen generation strategies. However, an efficient catalyst is required to reduce the energy barrier of the hydrogen evolution reaction (HER) [4]. So far, the most effective electrocatalysts for HER are Pt group materials, but the scarcity and high cost of these noble metals significantly limit their wide utilization [5]. Herein, the development of low-cost and efficient HER electrocatalysts based on earth-abundant species is of great importance [6,7,8]. 

Up to now, various non-precious metal-based materials (e.g., Ni, Co, Fe, Cu, W, and Mo) have been intensively synthesized as promising HER catalysts with high performance [9,10,11,12]. Among these alternatives, crystalline MoO_2_ has been identified as an excellent candidate owing to its good electric conductivity and high electrocatalytic activity [13,14,15]. It is noted that most of the reported non-precious electrocatalysts are based on crystalline compounds. In recent years, a growing class of amorphous materials have emerged as more efficient electrocatalysts compared with their crystalline counterparts [16,17,18,19,20]. However, the amorphous catalysts suffer from poor cycling stability caused by slow dissolution of the catalyst components during long-term test, thus resulting in easy degradation in the electro-activity [21,22]. To mitigate this critical problem, a large number of studies have shown that coupling different functional species can generate a strong synergistic effect to significantly improve the performance [22]. It is important to note that Ni-based electrocatalysts exhibit excellent HER catalytic activity in alkaline media due to the appropriate OH-Ni^2+δ^ (0 ≤ δ ≤ 1.5) bond strength [23,24]. Therefore, it is highly desirable and interesting to combine Ni-species with MoO_2_ to synergistically achieve substantial improvements in electro-activity and durability.

Herein, we highlight a Ni(P, O)_x_·MoO_x_ nanowire array which grows directly on a nickel foam support (Ni(P, O)_x_·MoO_x_ NA/NF) for a highly efficient electrocatalyst which exhibits preferable HER activity. The direct integration of nanowire array onto the Ni foam not only simplifies the electrode preparation processes, but also ensures the tight connection between electrode framework and active species, resulting in enhanced mechanical stability. In addition, the commercial nickel foam acts as a three-dimensional (3D) macroporous conductive substrate that facilitates facile charge transfer, electrolyte diffusion, and gas bubble release. Consequently, benefiting from the collaborative advantages of Ni-species and amorphous MoO_x_, the as-prepared Ni(P, O)_x_·MoO_x_ NA/NF electrode shows a remarkable electrocatalytic activity with a low overpotential of 59 mV to attain a current density of 10 mA cm^−2^ and superior stability for at least 24 h in an alkaline environment, thereby demonstrating a highly-efficient HER catalyst.

## 2. Results and Discussion

**Figure 1:**
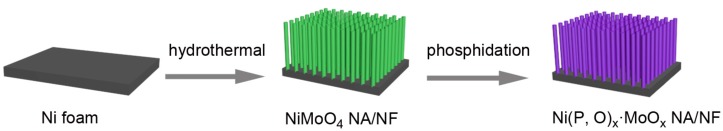
Schematic illustration of the formation processes of the Ni(P, O)_x_·MoO_x_ nanowire array which grows directly on a nickel foam support (Ni(P, O)_x_·MoO_x_ NA/NF).

**Figure 2:**
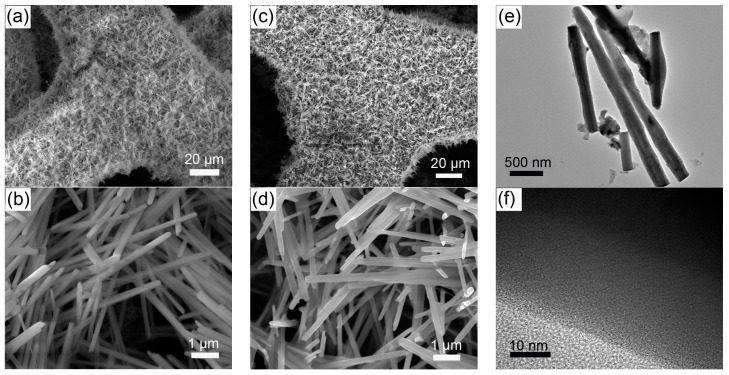
SEM images of (**a**,**b**) NiMoO_4_ NA/NF and (**c**,**d**) Ni(P, O)_x_·MoO_x_ NA/NF; (**e**) TEM and (**f**) HRTEM images of Ni(P, O)_x_·MoO_x_ NA/NF.

The self-supported Ni(P, O)_x_·MoO_x_ nanowire array is fabricated on a commercial Ni foam by a facile template-free hydrothermal process in combination with a subsequent in situ phosphorization treatment. Figure 1 schematically illustrates the typical two-step preparation process. In the first step, the Ni(P, O)_x_·MoO_x_ precursor (i.e., NiMoO_4_·xH_2_O) is grown on the 3D porous skeletons of the nickel foam by a hydrothermal reaction. In the second step, the Ni(P, O)_x_·MoO_x_ catalyst is obtained through a solid-state phosphorization process between the NiMoO_4_·xH_2_O precursor and NaH_2_PO_2_. The precursor is thermally transformed to crystalline NiMoO_4_ nanowire array supported on the Ni foam (NiMoO_4_ NA/NF), during which a simple dehydration reaction occurs. As can be seen from the scanning electron microscopy (SEM) image (Figure 2a), high-density NiMoO_4_ NA spreads uniformly over the nickel foam skeletons. A closer observation (Figure 2b) indicates that the diameter of the nanowire is about 210 nm, and the length is more than 6 μm. After phosphidation, the 1D nanowire array is maintained well from the precursors (Figure 2c,d), and the diameter of the Ni(P, O)_x_·MoO_x_ nanowires is similar to the NiMoO_4_. Transmission electron microscopy (TEM) was employed to further depict the as-prepared Ni(P, O)_x_·MoO_x_. Figure 2e shows the corresponding TEM image of Ni(P, O)_x_·MoO_x_ NA/NF, further identifying the preservation of the 1D morphology after phosphidation. The high-resolution TEM (HRTEM) image (Figure 2f) shows no obvious evidence of lattice fringes, suggesting that the as-synthesized Ni(P, O)_x_·MoO_x_ is amorphous or of poor crystallinity.

**Figure 3:**
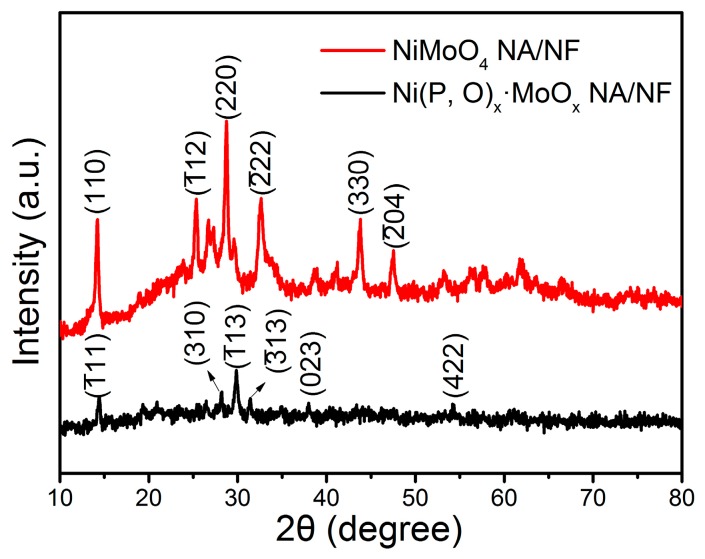
XRD pattern of the NiMoO_4_ NA/NF and Ni(P, O)_x_·MoO_x_ NA/NF.

The phase structure of the as-prepared samples was examined by X-ray diffraction (XRD) analysis. As shown in Figure 3, the distinct diffraction peaks with 2θ at around 14.3°, 25.4°, 28.9°, 32.6°, 43.9°, and 47.5° correspond to the (002), ($\bar{1}12$), (220), ($\bar{2}22$), (330), and ($\bar{2}04$) crystal planes of NiMoO_4_, respectively (JCPDS No. 86-0361) [25,26]. Compared with the crystalline NiMoO_4_, the Ni(P, O)_x_·MoO_x_ sample exhibits weak diffraction peaks, indicating that the phosphidation process results in a significant decrease in the crystallinity. The main peaks can be assigned to nickel phosphates (Ni_2_P_4_O_12_, JCPDS No. 76-1557). The absence of Mo-related peaks demonstrates that the Mo-based species are amorphous in the as-synthesized Ni(P, O)_x_·MoO_x_ NA/NF [27]. 

**Figure 4:**
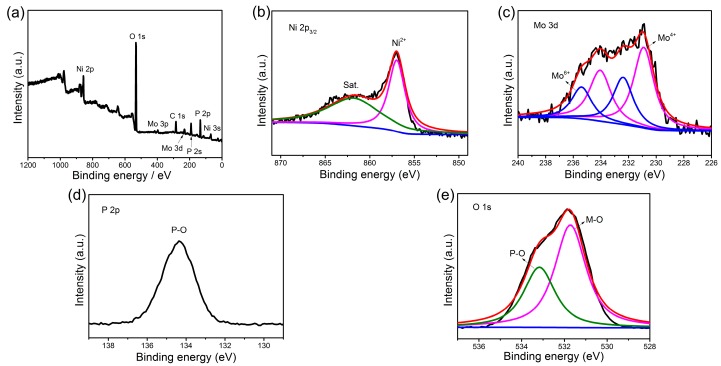
X-ray photoelectron spectroscopy (XPS) spectra of Ni(P, O)_x_·MoO_x_ NA/NF: (**a**) full scan; (**b**) Ni 2p_3/2_; (**c**) Mo 3d; (**d**) P 2p; and (**e**) O 1s. M–O: metal–oxygen.

X-ray photoelectron spectroscopy (XPS) measurement was carried out to investigate the surface composition and the oxidation state of the Ni(P, O)_x_·MoO_x_ NA/NF. The survey spectra show that the Ni(P, O)_x_·MoO_x_ NA/NF is composed of Mo, Ni, P, O elements (Figure 4a) and the atomic percentage of P in the product is 17.16%. The Ni 2p_3/2_ high-resolution spectrum (Figure 4b) exhibits two main peaks at binding energies of 856.9 and 862.1 eV, which can be assigned to the Ni–O bond and the satellite peak, respectively [28]. The Mo 3d spectrum of the Ni(P, O)_x_·MoO_x_ NA/NF (Figure 4c) can be resolved into two sets of peaks corresponding to the Mo^6+^ and Mo^4+^ species, and the ratio between the Mo^6+^ and Mo^4+^ in the composite is 0.57. The presence of Mo^4+^ species is probably attributed to the reduction of the Mo^6+^ precursor during phosphidation process [29]. For the profile of P 2p, the sample (Figure 4d) only shows a peak at a binding energy of 134.4 eV, which represents the P–O bond [30]. The high-resolution O 1s spectrum (Figure 4e) can be fitted into two peaks at 531.7 and 533.1 eV, which can be ascribed to the metal–oxygen (M–O) and P–O bonds, respectively [28].

**Figure 5:**
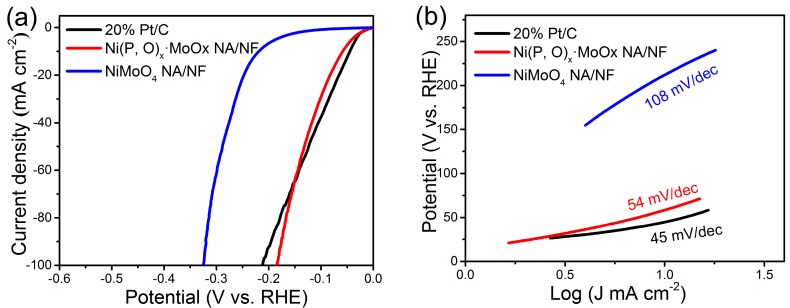
(**a**) Linear sweep voltammetry (LSV) polarization curves for hydrogen evolution reaction (HER) and (**b**) corresponding Tafel plots.

The HER performance of the Ni(P, O)_x_·MoO_x_ NA/NF was examined in 1 M KOH aqueous solution. For comparison, commercial Pt/C (20 wt % Pt/XC-72) and NiMoO_4_ NA/NF were also evaluated. Figure 5a shows the IR-corrected linear sweep voltammetry (LSV) curves. The Ni(P, O)_x_·MoO_x_ NA/NF electrode exhibits a low overpotential of 59 and 185 mV to reach a current density of 10 and 100 mA cm^−2^, respectively. In sharp contrast, the control NiMoO_4_ NA/NF electrode requires much higher overpotentials of 219 and 324 mV to achieve the same current densities. The lower overpotential of the Ni(P, O)_x_·MoO_x_ NA/NF electrode indicates a significant improvement in the HER catalytic property. Impressively, the overpotential is almost comparable to the commercial Pt/C electrode, demonstrating that the present electrode material may serve as a practical cathode for the high-efficiency production of hydrogen.

**Figure 6:**
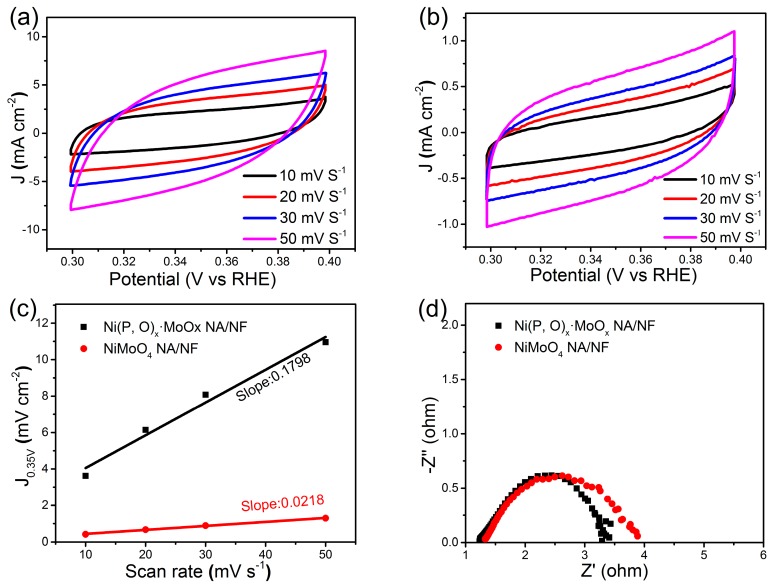
Cyclic voltammograms of (**a**) Ni(P, O)_x_·MoO_x_ NA/NF and (**b**) NiMoO_4_ NA/NF; (**c**) Scan rate-dependent current densities at 0.35 V (vs. reversible hydrogen electrode, RHE); and (**d**) Nyquist plots of Ni(P, O)_x_·MoO_x_ NA/NF and NiMoO_4_ NA/NF.

Figure 5b shows the corresponding Tafel plots. It is worth noting that the Tafel slope of Ni(P, O)_x_·MoO_x_ NA/NF is about 54 mV dec^−1^, which is only half of the control NiMoO_4_ NA/NF electrode (108 mV dec^−1^). This low Tafel slope indicates that the HER occurs on the Ni(P, O)_x_·MoO_x_ NA/NF electrode following the Volmer–Heyrovsky mechanism, and the rate-limiting step is the electrochemical recombination with an additional proton [9]. More importantly, the Ni(P, O)_x_·MoO_x_ NA/NF catalytic activity is superior to most Mo-based HER electrocatalysts reported so far (Table 1). In addition, the amount of catalytically active surface area on NiMoO_4_ NA/NF and Ni(P, O)_x_·MoO_x_ NA/NF electrodes are roughly estimated from the electrochemical double-layer capacitance (C_dl_) by measuring cyclic voltammetry (CV) curves at different scanning rates (Figure 6a,b). The determined C_dl_ for Ni(P, O)_x_·MoO_x_ NA/NF (89.9 mF cm^−2^) is much higher than NiMoO_4_ NA/NF (10.9 mF cm^−2^) (Figure 6c), suggesting a larger surface active area and more exposed active sites [10]. Figure 6d shows that the charge-transfer resistance of the Ni(P, O)_x_·MoO_x_ NA/NF electrode (3.4 Ω) is smaller than that of the NiMoO_4_ NA/NF (3.9 Ω), indicating rapid charge transfer. The large electro-active surface area along with the enhanced charge transfer kinetics of the Ni(P, O)_x_·MoO_x_ NA/NF are believed to be responsible for the associated higher HER catalytic activity.

**Figure 7:**
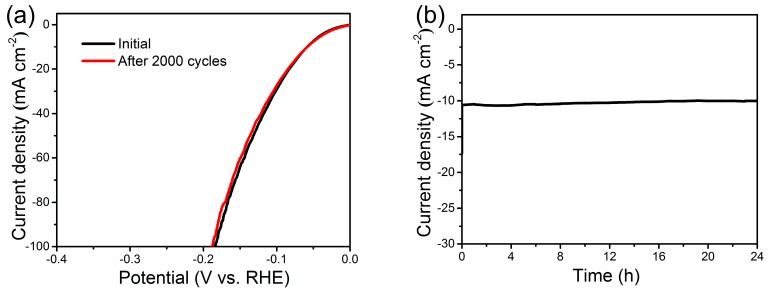
(**a**) Polarization curves for Ni(P, O)_x_·MoO_x_ NA/NF in 1 M KOH initially and after 2000 cycles at a scan rate of 50 mV s^−^^1^; (**b**) Time-dependent current density of Ni(P, O)_x_·MoO_x_ NA/NF at 60 mV (vs. RHE).

Good catalytic stability is of critical significance for an electrocatalyst when it comes to potentially practical implementation, particularly considering that the HER catalysts work in harsh environments [8]. The Ni(P, O)_x_·MoO_x_ NA/NF electrocatalyst was first evaluated via a recycling test using LSV method. As shown in Figure 7a, the LSV curves are almost overlapped with a slight loss of the cathodic current densities, indicating a negligible active degradation before and after 2000 scanning cycles. The excellent cycling stability is further validated by the time dependence of the current density curve at a constant overpotential of 60 mV (Figure 7b). The Ni(P, O)_x_·MoO_x_ NA/NF manifests a stable catalytic current over 24 h, confirming the long-term durability of the electrocatalytic activity.

It is believed that the high alkaline HER performance of the Ni(P, O)_x_·MoO_x_ NA/NF can be attributed to the combination of compositional and geometric advantages: (1) Ni^2+^ is of great benefit for the adsorption of hydroxyl species, while amorphous MoO_x_ provides catalytically active sites for the adsorption of H* intermediate and further facilitates the subsequent formation of H_2_. The synergistic cooperation greatly reduces the energy barriers of the initial water decomposition and the subsequent step of H_2_ generation. (2) Coupling Ni-species and amorphous MoO_x_ generates a strong synergistic effect to significantly improve the stability. (3) The nanowire array offers a larger surface active area with more exposed active sites. (4) The 3D porous and conductive nickel foam not only effectively increases the contact area between active catalyst and electrolyte, but also serves as a robust skeleton to provide strong mechanical adhesion and electric connection to the nanowire array, thereby ensuring facile charge and mass transport, gas bubble release, and good electrode structure for long-term test.

## 3. Materials and Methods

### 3.1. Synthesis of Ni(P, O)_x_·MoO_x_ NA/NF

All chemical reagents were of analytical grade and used as received without further purification. The Ni foam with a thickness of 1.6 mm and dimensions of 2 × 4 cm^2^ was sonicated in diluted hydrochloric acid (1 M), acetone, deionized water, and ethanol for 10 min, respectively. In a typical synthetic process, 1 mmol Ni(NO_3_)_2_·6H_2_O and 1 mmol Na_2_MoO_4_·2H_2_O were dissolved in 30 mL H_2_O to form a clear solution. Subsequently, the solution and purified Ni foam were transferred into a 50 mL Teflon-lined stainless autoclave, which was sealed and heated at 160 °C for 6 h in an oven. After the reaction, the resulting light-green Ni foam was rinsed with deionized water and ethanol, then the sample was dried at 60 °C for overnight.

In the next step, the obtained NiMoO_4_·xH_2_O NA/NF precursor and 10 mmol NaH_2_PO_2_ were placed at two separate positions of the tube furnace with the NaH_2_PO_2_ at the upstream side. Subsequently, the samples were heated at 400 °C for 120 min with a ramp rate of 2 °C min^−1^ under flowing nitrogen. After cooling to room temperature naturally, the resulting electrode was obtained. The mass loading of the as-prepared Ni(P, O)_x_·MoO_x_ NA/NF on the Ni foam was ~5.4 mg cm^−2^. The synthesis of NiMoO_4_ NA/NF was the same as Ni(P, O)_x_·MoO_x_ NA/NF, just without NaH_2_PO_2_.

### 3.2. Material Characterization

The crystallographic phase of the products was examined by X-ray diffraction (XRD) with Cu Kα radiation (λ = 0.15418 nm) (X’Pert Pro MPD, Philips, Almelo, The Netherlands). The morphology was characterized by field emission scanning electron microscopy (FE-SEM, FEI Nano SEM 450, FEI, Portland, OR, USA) and transmission electron microscopy (TEM, FEI Tecnai F30G2, FEI, Portland, OR, USA). The surface chemistry and elemental analysis of the sample were characterized by X-ray photoelectron spectroscopy (XPS, ESCALAB 250Xi, Thermo Scientific, Waltham, MA, USA).

### 3.3. Electrochemical Measurements

The catalytic performances of the electrocatalysts were investigated by using an electrochemical workstation (Solartron 1260 + 1287, Bognor Regis, West Sussex, UK) in a three-electrode system. The Ni(P, O)_x_·MoO_x_ NA/NF was used as the working electrode; a graphite rod and the saturated calomel electrode (SCE) were used as the counter and reference electrode, respectively. All of the finial potentials were calibrated to a reversible hydrogen electrode (RHE). The polarization curves were corrected with IR compensation. The working electrodes were activated before the measurement by cyclic voltammetric scans with a scan rate of 50 mV s^−^^1^. The HER performances of the obtained electrocatalysts were tested from 0.2 to −0.4 V (vs. RHE) in 1 M KOH aqueous solution by LSV with a scanning rate of 2 mV s^−1^. Electrochemical impedance spectroscopy (EIS) was carried out at −0.2 V (vs. RHE) over a frequency range from 100 kHz to 0.01 Hz with a 10 mV AC dither. To determine the catalytically active surface area of the products, the electrochemical double-layer capacitance (C_dl_) of the electrodes was estimated by using CV method in a non-Faradaic range of 0.3–0.4 V (vs. RHE) at various scan rates. A linear relationship between the current densities at 0.35 V (vs. RHE) and scan rate can be plotted to obtain C_dl_, the value of which is half of the resulting slope. The catalytically active surface area of different electrocatalysts can be directly compared by the C_dl_ values, because the C_dl_ is in proportion to the active surface area [28].

## 4. Conclusions

In summary, a novel Ni(P, O)_x_·MoO_x_ nanowire array supported on a Ni foam was prepared via a facile approach. Because of the synergistic effect of the Ni-species and amorphous MoO_x_, the as-prepared catalyst exhibits excellent electrocatalytic performance in an alkaline media, including a low overpotential of 59 mV at 10 mA cm^−2^, a small Tafel slope of 54 mV dec^−1^, and long-term stability. The enhanced electrocatalytic performance demonstrates the advantageous combination of compositional and geometric factors. The present work also provides an avenue to fabricating low-cost alkaline electrocatalysts for practical implementation.

## Figures and Tables

**Table 1:** Comparison of HER performance for Ni(P, O)_x_·MoO_x_ NA/NF with Mo-based electrocatalysts.

Catalyst ^[a]^	Overpotential at j = 10 mA cm^−2^ (mV)	Tafel Slope (mV dec^−1^)	Electrolyte	Reference
MoO_2_@PC-RGO	64	41	0.5 M H_2_SO_4_	[15]
MoP/Ni_2_P/NF	75	100	1 M KOH	[28]
Ni/Mo_2_C	179	101	1 M KOH	[31]
NiMoN-550	89	79	1 M KOH	[32]
Mo_2_C@NC	60	60	1 M KOH	[33]
MoP NA/CC	80	83	1 M KOH	[34]
MoS_2_/MoO_2_	240	76	0.5 M H_2_SO_4_	[35]
MoO_2_/RGO	—	68	0.5 M H_2_SO_4_	[36]
MoP|S	64	50	0.5 M H_2_SO_4_	[37]
Ni(P, O)_x_·MoO_x_ NA/NF	59	54	1 M KOH	This work

^[a]^ PC-RGO: phosphorus-doped carbon-reduced grphene oxide; NC: nitrogen-rich carbon; CC: carbon cloth.
